# Harnessing natural feed additives for sustainable production and economics: the role of *Thymus vulgaris* L. oil as an antimicrobial agent and a growth promoter in improving production and health of broiler chickens

**DOI:** 10.3389/fimmu.2025.1695478

**Published:** 2025-12-05

**Authors:** Ahmed M. Saied, Adel I. Attia, Fayiz M. Reda, Mohamed S. El-Kholy, Mohammed A. Al-Badwi, Mahmoud Azzam, Alessandro Di Cerbo, Mahmoud Alagawany, Khaled A. El-Tarabily, Ayman G. EL Nagar

**Affiliations:** 1 Poultry Department, Faculty of Agriculture, Zagazig University, Zagazig, Egypt; 2 Department of Animal Production, College of Food and Agricultural Sciences, King Saud University, Riyadh, Saudi Arabia; 3 School of Biosciences and Veterinary Medicine, University of Camerino, Matelica, Italy; 4 Department of Biology, College of Science, United Arab Emirates University, Al Ain, United Arab Emirates; 5 Department of Animal Production, Faculty of Agriculture at Moshtohor, Benha University, Benha, Egypt

**Keywords:** broilers, economics, growth, intestinal morphology, microbiota, organic poultry, thyme

## Abstract

**Introduction:**

This study examined the effects of adding thyme oil (TO) to broiler diets on growth performance, intestinal health, immune organ indices, blood biochemical parameters, and economic feasibility.

**Methods:**

In a completely randomized block design, five groups of 1-day-old broiler chicks (n=300) with five replicates, each consisting of 12 chicks, were used. A basal diet (BD) was supplied to the control group, whereas the other groups received BD supplemented with 75, 150, 225, and 300 mg/kg of TO.

**Results and discussion:**

The findings indicated that TO improved final body weight (BW), total body weight gain (BWG) and the feed conversion ratio (FCR), without affecting feed intake (FI). Groups fed 150, 225, and 300 mg/kg of TO showed higher levels of digestive enzymes (*P*<0.05) than the control. Supplementation with TO elevated immune organ indices, with statistical significance (*P*<0.05) noted in birds receiving 150, 225, and 300 mg TO/kg for the spleen, thymus, and bursa of Fabricius indices, compared with the control group. Moreover, total protein, globulin, immunoglobulin (IgM and IgG), and complement component 3 were significantly (*P<0.05*) higher by TO at 150, 225, and 300 mg/kg than at 0 and 75 mg/kg. Blood Ca and Mg were significantly (*P*<0.05) increased by the application of 150, 225, and 300 mg TO/kg, whereas the Fe level rose (*P*<0.05) by 75 and 150 mg/kg compared with the control. TO at 150, 225, and 300 mg/kg increased hemoglobin (Hb), counts of white blood cells (WBCs), and red blood cells (RBCs) compared with the control. The application of TO decreased (*P<0.05*) aspartate aminotransferase (AST) levels in a dose-dependent manner. Creatinine and urea levels were significantly decreased by TO at 225 and 300 mg/kg compared with the control. Lipid peroxidation was significantly (*P<0.05*) decreased by TO supplementation (150, 225, and 300 mg/kg), whereas superoxide dismutase (SOD), total antioxidant capacity (TAC), catalase (CAT), and reduced glutathione (GSH) were significantly increased by the application of 150, 225, and 300 mg TO/kg compared with the control. Lower levels of total cholesterol, triglycerides, low-density lipoprotein (LDL), and very low-density lipoprotein (VLDL) were observed at 75, 150, 225, and 300 mg TO/kg compared with 0 mg/kg. In comparison, higher high-density lipoprotein (HDL) levels were observed for 225 and 300 mg of TO/kg compared with the control. The net revenue and economic efficiency of TO-supplemented treated broilers were improved during the experiment (1–6 weeks of age). The best values of economic indices were observed in the group receiving 150 mg/kg of TO. Collectively, supplementation with dietary TO enhanced growth performance, intestinal health, immune system development and function, liver and kidney function, and antioxidant capacity, regulated the lipid profile, and improved economic feasibility.

## Introduction

The global reduction of antibiotics in poultry has driven interest in natural alternatives, including microbial and phytogenic sources (e.g., probiotics, prebiotics, and essential oils), which exhibit growth-promoting, antioxidant, antimicrobial, and immunostimulant effects in animals (1–8). Thyme (*Thymus vulgaris* L.) is a Mediterranean herb used for food, medicine, and decoration due to its high bioactive component content and ethnopharmacological significance (5–8).

Thyme plants’ flowers, leaves, and oil can help people with various ailments. It exerts antihypertensive (9), anticancer, antiviral, antifungal (10), and antimicrobial and anti-inflammatory properties (11). Additionally, thyme is a viable source of natural feed additive because it increases food stability and reduces lipid oxidation over the course of food storage (animal products) (12). Thyme oil (TO) stability during industrial scalability depends on formulation and processing. This stability is critical for bird efficacy and palatability, as it is sensitive to temperature, oxygen, and light, which can lead to decreased stability, peroxidation, and reduced feed intake (12).

Thymol and carvacrol, the key components of thyme essential oil, can improve poultry health (5, 12, 13). Moreover, thyme extract (1 mL/L drinking water) alleviates growth impairment in broilers fed low-protein diets (13). Oke et al. (14) reported that thyme can help birds under oxidative stress by increasing their antioxidant levels.

Increased serum antioxidant capacity and decreased mortality rate were observed in broiler diets supplemented with thymol (15). Furthermore, thyme has antimicrobial and antiviral characteristics that impact numerous microorganisms (5, 15). The application of thyme essential oil or thyme powder in broiler feed elicited immune system stimulation (16) and stress reduction (17).

Data on the use of TO on intestinal architecture, digestive enzymes, and the economic efficiency of poultry are scarce, and previous findings (15, 17–19) on TO supplementation in broiler chicken diets yielded contradictory conclusions. Therefore, the objective of the present study was to explore the impacts of dietary TO supplementation at graded levels (75, 150, 225, and 300 mg/kg) on a wide range of measurements including intestinal digestive enzymes, cecal microbiota, immunity, antioxidant status, carcass characteristics, some hematological parameters, blood minerals, and blood markers of liver and kidney function, besides the growth performance indices and economic efficiency. The trial was terminated at 42 days of age, which corresponds to the typical slaughter age for commercial broilers under intensive management systems. This timing ensures the assessment of TO effects throughout the birds’ entire commercial growing cycle, including both the starter and finisher phases.

## Materials and methods

This study was conducted at a private poultry farm in San El Hagar, Sharkia Governorate, Egypt, in accordance with the ethical guidelines of the Poultry Department at Zagazig University, Zagazig, Egypt. All laboratory analyses, experimental procedures, and protocols adhered to the ethical standards set by the Committee of Animal Care and Welfare at Zagazig University, under Ethical Approval Number ZU-IACUC/2/F/313/2023.

Birds were monitored daily for health, including mortality and morbidity, and humane endpoints were applied. Any animal showing severe signs of distress or illness was treated or humanely euthanized following standard protocols.

### Phytoextracts tested and their characteristics

The TO used in this study was obtained from El Hawag Company for Natural Oils, located in Cairo, Egypt. Gas chromatography-mass spectrometry (GC-MS) analysis identified the primary compounds of TO as follows: 48% thymol, 17.5% γ-terpinene, 13.6% p-cymene, and 11% carvacrol.

### Experimental birds and design

Five groups of unsexed Arbor Acres 1-day-old broiler chicks (n=300; 5 replicates of 12 chicks each) were used. Five dietary treatments were provided: a control diet (basal diet) and four test groups with varying TO concentrations (75, 150, 225, and 300 mg/kg diet). The replicates were kept apart in 100 × 120-cm pens. Birds were randomly distributed into pens at the beginning of the experiment to ensure homogeneity in body weight (BW) among groups. Pens were subsequently assigned to dietary regimens at random to eliminate bias and confounding factors.

A nutritionally balanced basal diet was carefully formulated to meet the nutritional requirements of broiler chicks throughout their growth period, from days 1 to 42, in accordance with the National Research Council guidelines (20). The detailed chemical composition and nutrient content of the basal diet are presented in **Table 1**. The same care and hygienic circumstances were used to raise each bird.

**Table 1:** Feed ingredients of the basal diet during the stages of broiler growth.

Ingredients (% as fed)	Starter ration (days 1-21)	Finisher ration (days 22-42)
Maize (8.5% crude protein)	53.03	59.21
Soybean meal (44% crude protein)	35.00	27.00
Maize gluten meal (62% crude protein)	5.00	5.00
Soybean oil	2.90	4.82
Limestone	1.40	1.37
Di-calcium phosphate	1.50	1.55
Salt	0.30	0.30
*Premix	0.30	0.30
L-Lysine	0.15	0.15
DL-Methionine	0.12	–
Choline chloride	0.30	0.30
**Nutritional composition		
Metabolizable energy (kcal/kg)	3000	3200
Crude protein (%)	23.01	20.01
Calcium (%)	1.02	1.00
Non-phytate phosphorus (%)	0.45	0.45
Lysine (%)	1.32	1.10
Total sulfur amino acids (%)	0.92	0.72

^*^Provides per kg of diet: vitamin A, 12,000 I.U; vitamin D3, 5,000 I.U; vitamin E, 130.0 mg; vitamin K3, 3.605 mg; vitamin B1 (thiamin), 3.0 mg; vitamin B2 (riboflavin), 8.0 mg; vitamin B6, 4.950 mg; vitamin B12, 17.0 mg; niacin, 60.0 mg; D-biotin, 200.0 mg; calcium D-pantothenate, 18.333 mg; folic acid, 2.083 mg; manganese, 100.0 mg; iron, 80.0 mg; zinc, 80.0 mg; copper, 8.0 mg; iodine, 2.0 mg; cobalt, 500.0 mg; and selenium, 150.0 mg. **According to the National Research Council guidelines (20).

Chicks were reared with unlimited accessibility to water and feed. During the experiment period, the birds were raised at a standard ambient temperature (decreasing by 3 °C/week from 30°C to 24°C) with a relative humidity of 60%–70%. There was an artificial light source that gave light for (23L: 1D) h every day.

### Growth performance and organ weights

Chicks’ weight and feed intake (FI) were assessed biweekly at 1, 14, 28, and 42 days of age. Calculations were made for body weight gain (BWG) and the feed conversion ratio (FCR, FI/BWG). On day 42, five birds were randomly selected from each treatment, weighed, and slaughtered in accordance with Islamic practices. Individual weights in grams were recorded for the edible giblets (heart, gizzard, and liver) and lymphoid organs (spleen, bursa, and thymus). Weights were determined for the dressing (carcass + total edible giblets) and the total edible giblets.

### Blood analysis

To test the effect of TO supplementation on hematology and blood chemistry, five birds were randomly selected from each treatment for blood sampling. The birds were humanely slaughtered following standard practices, followed by cervical dislocation. Blood samples were collected from the slaughtered animals to assess relevant biochemical parameters.

For laboratory analysis, blood samples were collected in ethylenediaminetetraacetic acid (EDTA) tubes (Vacutest Kima, Arzergrande PD, Italy). In whole blood, hemoglobin (Hb) content and the counts of red and white blood cells (RBCs and WBCs) were determined according to Dein (21), within 1 h of blood collection. Furthermore, blood glucose was measured in the fresh blood collected during the procedure using a home glucose monitor (Accu-Chek Active, Roche Diagnostics, Basel, Switzerland) with disposable test strips.

The RBC (10^6^/µL) and WBC (10^3^/µL) counts were carried out using a Neubauer improved hemocytometer (Paul Marienfeld GmbH and Co., KG, Lauda-Königshofen, Germany) after appropriate dilution with Natt-Herricks solution (22). The Hb content was determined by the acid hematin (Sahli’s) method as described by Bain et al. (23). Measurements were performed using a UV-Vis spectrophotometer (Shimadzu UV-1800, Shimadzu Corporation, Kyoto, Japan) at 540 nm with a commercial acid hematin reagent (Sigma-Aldrich Chemie GmbH, Taufkirchen, Germany).

The remaining blood samples were subjected to centrifugation for 10 min at 5,000 rpm, and the blood plasma was separated and stored at −20°C for further biochemical analysis. Using commercial kits according to the manufacturer’s regulations (Labtest Diagnóstica S.A, Lagoa Santa, Brazil), total protein (g/dl, Ref.: 1085; wavelength 545 nm), albumin (g/dl, Ref.: 1007; wavelength 630 nm), creatinine (mg/dl, Ref.: 1010; wavelength 510 nm), urea (mg/dl, Ref.: 1013; wavelength 340 nm), uric acid (mg/dl, Ref.: 140; wavelength 520 nm), triglycerides (mg/dl, Ref.: 87; wavelength 505 nm), total cholesterol (mg/dl, Ref.: 1082; wavelength 500 nm), and high-density lipoprotein (HDL) cholesterol (mg/dl, Ref.: 13; wavelength 500 nm) along with the activity of liver aspartate aminotransferase (AST) (IU/L, Ref.: 1009; wavelength 340 nm) and alanine aminotransferase (ALT) (IU/L, Ref.: 1008; wavelength 340 nm) were measured on an automated chemistry analyzer (Mindray Bio-Medical Electronics Co., Shenzhen, China).

By deducting plasma albumin from plasma total protein, plasma globulin was computed. Triglycerides were divided by five to determine plasma very low-density lipoprotein (VLDL) cholesterol (mg/dl). HDL and VLDL were subtracted from total cholesterol to determine low-density lipoprotein (LDL) cholesterol (mg/dl) (24).

Blood plasma levels of minerals were conducted by a UV-Vis spectrophotometer (Biochrom Libra S22, Biochrom Ltd., Cambridge, UK) using specific kits of Spectrum Diagnostics company (Obour City, Cairo, Egypt) for Mg (mg/dl, REF: 285002; wavelength 630 nm), Fe (μmol/L, REF: 269002; wavelength 623 nm), Ca (mg/dl, REF: 226002; wavelength 578 nm), and P (mg/dl, REF: 294002; wavelength 340 nm).

Plasma concentrations of key oxidative stress and antioxidant markers were spectrophotometrically measured (Shimadzu Corporation) using specific commercial kits (Abcam Limited, Cambridge, UK) for reduced glutathione (GSH; mg/dl, Catalog number 239727; wavelength 450 nm), superoxide dismutase (SOD; U/mL, Catalog number ab65354; wavelength 450 nm), total antioxidant capacity (TAC; ng/mL, Catalog number ab65329; wavelength 570 nm), malondialdehyde (MDA; nmol/mL, Catalog number ab118970; wavelength 570 nm), and glutathione S-transferases (GST; mg/dl, Catalog number ab65326; wavelength 340 nm). Catalase (CAT; ng/mL, Catalog number ab83464; wavelength 570 nm) activity was assessed using a UV-based method as described by Mueller et al. (25).

Additionally, immunological parameters were measured in plasma using a sandwich ELISA kit (MyBioSource, San Diego, USA), adapted from the work of Bianchi et al. (26) for immunoglobulin G (IgG) (mg/dl, Catalog number MBS260043), immunoglobulin M (IgM) (mg/dl, Catalog number MBS706158), and immunoglobulin A (IgA) (mg/dl, Catalog number MBS564152), along with blood values of complement component 3 (C3-mg/dl, Catalog number MBS013330) and lysozyme activity (mg/dl, Catalog number MBS2019829) according to the manufacturers’ instructions using the BioTek ELx800 microplate reader (Marshall Scientific, Hampton, USA).

### Assay of digestive enzymes

Small intestine digesta samples were collected from each slaughtered bird by squeezing the intestinal tract. Each sample was weighed, diluted (1:10) with ice-cold phosphate-buffered saline (pH 7.2, Sigma-Aldrich), homogenized using T18 digital ULTRA-TURRAX^®^ (IKA, Staufen, Germany), and centrifuged at 5,000 × *g* for 20 min at 4 °C using an Eppendorf 5810R centrifuge (Eppendorf SE, Hamburg, Germany).

The supernatant was stored at 4°C for analysis using a sandwich ELISA kit (MyBioSource Company, San Diego, USA) for amylase (Catalog number MBS269117), lipase (Catalog number MBS7222144), and protease (Catalog number MBS105713) using the BioTek ELx800 microplate reader (Marshall Scientific).

### Cecal microbiota

Cecal content (1 g) was collected from five birds per treatment and individually placed into 250-mL Erlenmeyer flasks containing 90 mL of sterile peptone (0.1%) and saline solution (0.85% NaCl). The mixture was thoroughly blended to ensure uniform distribution. Microbial analysis was conducted using selective agar media (Becton Dickinson, New Jersey, USA) to determine the counts of *E. coli* (MacConkey agar), *Salmonella* (XLD agar), lactic acid bacteria (de Man, Rogosa, and Sharpe (MRS) agar), total yeasts and molds (TYMC; Sabouraud dextrose agar), and total bacterial count (TBC; plate count agar). Plates were incubated in an IN30 incubator (Memmert GmbH, Schwabach, Germany) at appropriate temperatures per species. Methods were based on those of Xia et al. (27).

### Histopathological examination

After the trial was completed, five birds from each experimental group were randomly selected and humanely slaughtered for histopathological examination. Postmortem, the intestine, liver, bursa of Fabricius, and spleen were carefully excised and immediately immersed in 10% neutral buffered formalin solution (NBF) for tissue fixation.

Fixation was performed for 24 h at room temperature to preserve tissue architecture and prevent autolysis and degradation. Following fixation, tissue samples were subjected to a standard dehydration protocol through a graded series of ethanol concentrations (70%, 80%, 90%, 95%, and absolute alcohol), ensuring gradual removal of water from the tissues. Dehydrated tissues were then cleared in xylene (xylol) to replace the alcohol and to prepare them for embedding.

Subsequently, tissues were infiltrated and embedded in paraffin wax to obtain solid paraffin blocks suitable for microtomy. Thin paraffin sections with a thickness of 5 µm were obtained using a rotary microtome (Leica RM 2155, Leica Biosystems, London, UK). For histopathological evaluation, the tissue sections were stained using the routine hematoxylin and eosin (H&E) staining method (28). The stained slides were then examined under a light microscope (Olympus CX43, Olympus Corp., Tokyo, Japan) to assess histological architecture and detect any pathological alterations in the examined organs.

To evaluate the histomorphometric characteristics of the intestinal villi, stained tissue sections were examined under a light microscope (Olympus CX43) with a calibrated eyepiece micrometer. The following parameters were measured:

- Villus length (VL, μm): The vertical distance from the tip of the villus to the junction between the villus and crypt.
- Villus width (VW, μm): The horizontal width measured at the mid-point of the villus.
- Crypt depth (CD, μm): The depth of the invagination between two adjacent villi.
- Villus height to crypt depth ratio (VL/CD): Calculated by dividing villus length by crypt depth.
- Absorption surface area (ASA, mm²): Calculated by multiplying VL (mm) by VW (mm).

### Economic analysis

The NR (net revenue) was divided by all costs to compute the EE (economic efficiency). The formulas were used to calculate NR and EE. Net income was calculated as the cost of each 1 kg of meat produced plus all feed expenses.

The EE was calculated by dividing NR by the feed input. Labor, housing, and veterinary care costs were not included because they were the same across all treatments.

### Statistical analysis

Before analysis of variance (ANOVA), the Shapiro–Wilk W test was used to assess normality, and Levene’s test was used to assess homogeneity of variances. Also, logarithmic or arcsine transformations were applied to all percentage values.

ANOVA was conducted using the statistical model: Y_ij_ = *μ* + α_i_ + e_ij_

where Y_ij_ represents the observation, *μ* is the overall mean, αi is the fixed effect of treatment (1–5), and e_ij_ is the random error.

Tukey’s test identified significant differences among group means. Statistical analyses were performed using SAS software (version 9.4, SAS Institute Inc., Cary, NC, USA). Results were considered statistically significant at *P*<0.05 and presented as means ± SEM.

## Results

### Essential oils and bioactive components in TO

The GC-MS analysis outputs for TO, including area percentage and retention time, are shown in **Table 2**. The most abundant components in TO were thymol (48.00%), eucalyptol (24.30%), γ-terpinene (17.50%), p-cymene (13.50%), carvacrol (11.00%), isothymol methyl ether (6.15%), β-linalool (4.59%), and β-myrcene (3.35%), as well as 4-carene, isoborneol, and caryophyllene (ranged between 2.45% and 2.53%). These bioactive components and natural antioxidants play a key role in protecting cells from free radical-induced oxidative damage, which can negatively affect the immune system and public health.

**Table 2:** Bioactive chemical components identified in thyme oil using gas chromatography–mass spectrometry (GC-MS) analysis.

Bioactive chemical constituents	Retention time (RT) (min)	Area %
β-Myrcene	9.16	3.35
4-Carene	9.90	2.53
p-cymene	10.26	13.50
Eucalyptol	10.40	24.30
γ-Terpinene	11.23	17.50
β-Linalool	12.52	4.59
Isoborneol	14.60	2.50
Terpinen-4-ol	14.90	2.20
Isothymol methyl ether	16.90	6.15
Carvacrol	18.45	11.00
thymol	18.50	48.00
Thymyl acetate	20.07	0.45
Caryophyllene	21.90	2.45

### Effect of dietary supplementation with TO on growth performance of broiler chickens

**Figure 1:**
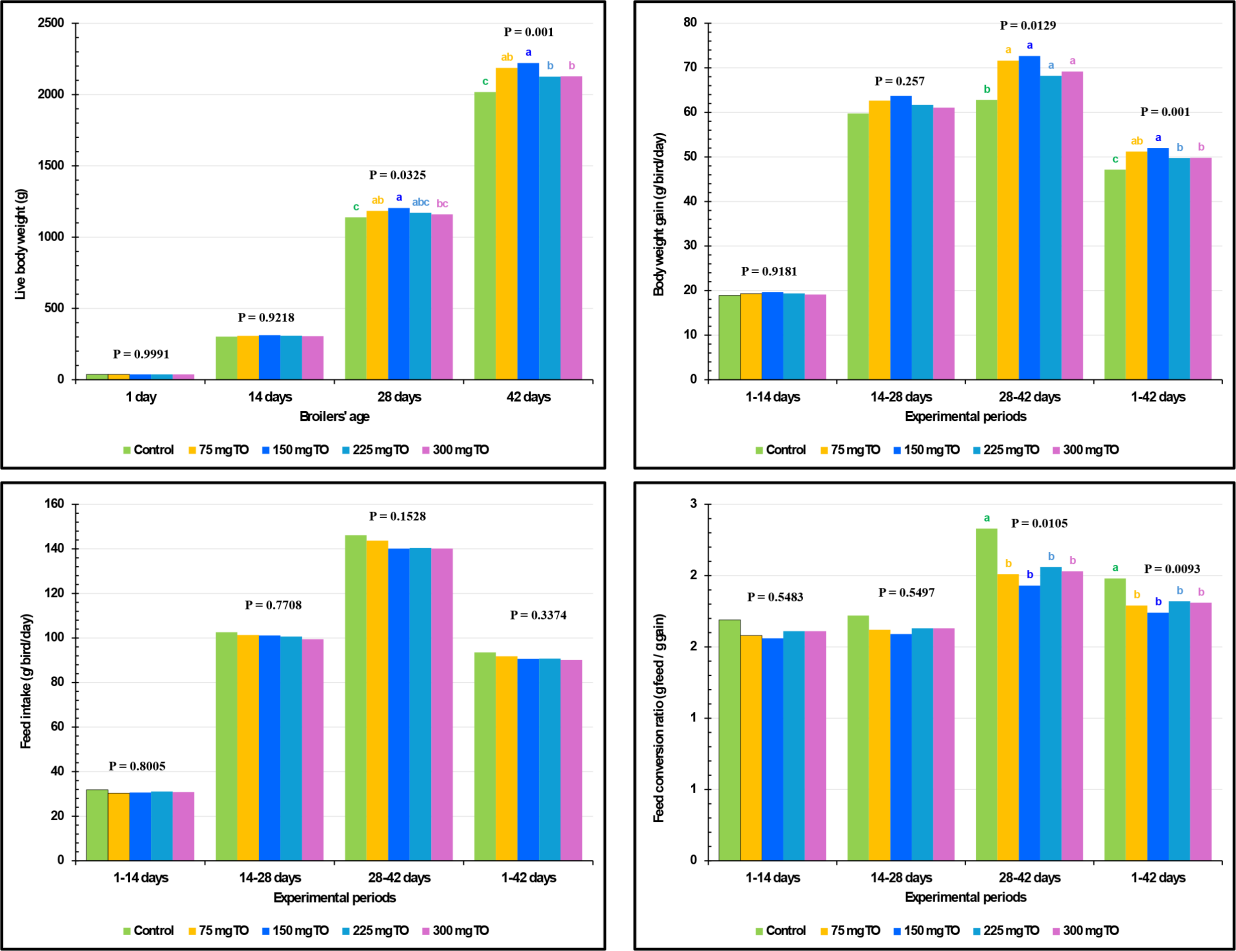
Impact of dietary supplementation with varying concentrations of thyme oil (TO) on the growth performance of broiler chickens.

The growth performance measurements of experimental birds are displayed in **Figure 1**. All concentrations of TO significantly boosted the BWG between 28 and 42 days of age (*P =* 0.0129) and overall BWG (1–42 days) (*P* = 0.0010) compared with control chickens (**Figure 1**).

The use of 150 mg/kg of TO was found to induce the highest final BW (*P* = 0.0010) followed by the 75 mg/kg diet, and then 225 and 300 mg/kg. Remarkably, at every studied interval, TO supplementation did not (*P*>0.05) affect FI. The addition of TO at all levels significantly improved FCR compared with the control birds during the intervals of 28-42 (*P* = 0.0105) and 1-42 (*P =* 0.0093) days (**Figure 1**).

### Effect of dietary supplementation with TO on digestive enzymes of broiler chickens

Protease, amylase, trypsin, and lipase levels rose in TO-supplemented groups, depending on the dose (**Table 3**). Beginning at the 150-mg/kg diet, TO dietary inclusion induced higher protease (*P<*0.0001), amylase (*P<*0.0001), trypsin (*P =* 0.0043), and lipase (*P =* 0.0001) levels than the control group (**Table 3**).

**Table 3:** Effect of dietary supplementation with varying concentrations of thyme oil on the digestive enzymes of broiler chickens.

Parameters	Thyme oil level (mg/kg diet)					SEM	*P* value
	0	75	150	225	300		
Protease (U/L)	0.25 *e*	1.46 *d*	2.76 *c*	4.23 *b*	5.28 *a*	0.371	<0.0001
Amylase (U/L)	126.45 *c*	143.41 *c*	216.46 *b*	227.15 *ab*	241.53 *a*	9.417	<0.0001
Trypsin (U/L)	85.97 *c*	97.50 *c*	118.69 *b*	124.53 *ab*	138.24 *a*	6.902	0.0043
Lipase (U/L)	18.97 *c*	21.64 *c*	36.78 *b*	52.81 *a*	47.49 *a*	3.394	0.0001

Values with the same letter within a row are not significantly (*P*>0.05) different according to Tukey’s test.

### Effect of dietary supplementation with TO on the cecal microbiota of broiler chickens

**Table 4** shows that dietary TO inclusion in broiler chicken diets at all doses significantly reduced counts of total bacteria (*P* = 0.0003), total mold and yeast (*P* = 0.0002), *Salmonella* (*P*<0.0001), and *E. coli* (*P*<0.0001) compared with the control.

**Table 4:** Effect of dietary supplementation with varying concentrations of thyme oil on cecal microbiota of broiler chickens.

Microbiological counts (Log_10_ CFU/g)	Thyme oil level (mg/kg diet)					SEM	*P* value
	0	75	150	225	300		
Total bacterial count	5.55 *a*	5.34 *b*	5.30 *b*	5.21 *d*	5.23 *cd*	0.032	0.0003
Total yeast and mold count	4.95 *a*	4.62 *b*	4.61 *bc*	4.63 *b*	4.54 *c*	0.035	0.0002
*Escherichia coli*	5.91 *a*	5.49 *b*	4.85 *c*	4.82 *c*	4.56 *d*	0.051	<0.0001
Lactic acid bacteria	3.73 *c*	3.71 *c*	3.87 *b*	4.05 *a*	3.92 *b*	0.046	0.0034
*Salmonella*	3.18 *a*	2.70 *b*	2.35 *c*	2.25 *c*	2.07 *d*	0.053	<0.0001

Values with the same letter within a row are not significantly (*P*>0.05) different according to Tukey’s test.

TO supplementation reduced the *E. coli* and *Salmonella* counts in a dose-dependent way (**Table 4**). However, compared with the control, TO supplementation significantly increased the lactic acid bacterial count (*P* = 0.0034) at 150, 225, and 300 mg/kg.

### Histopathological findings

**Figure 2:**
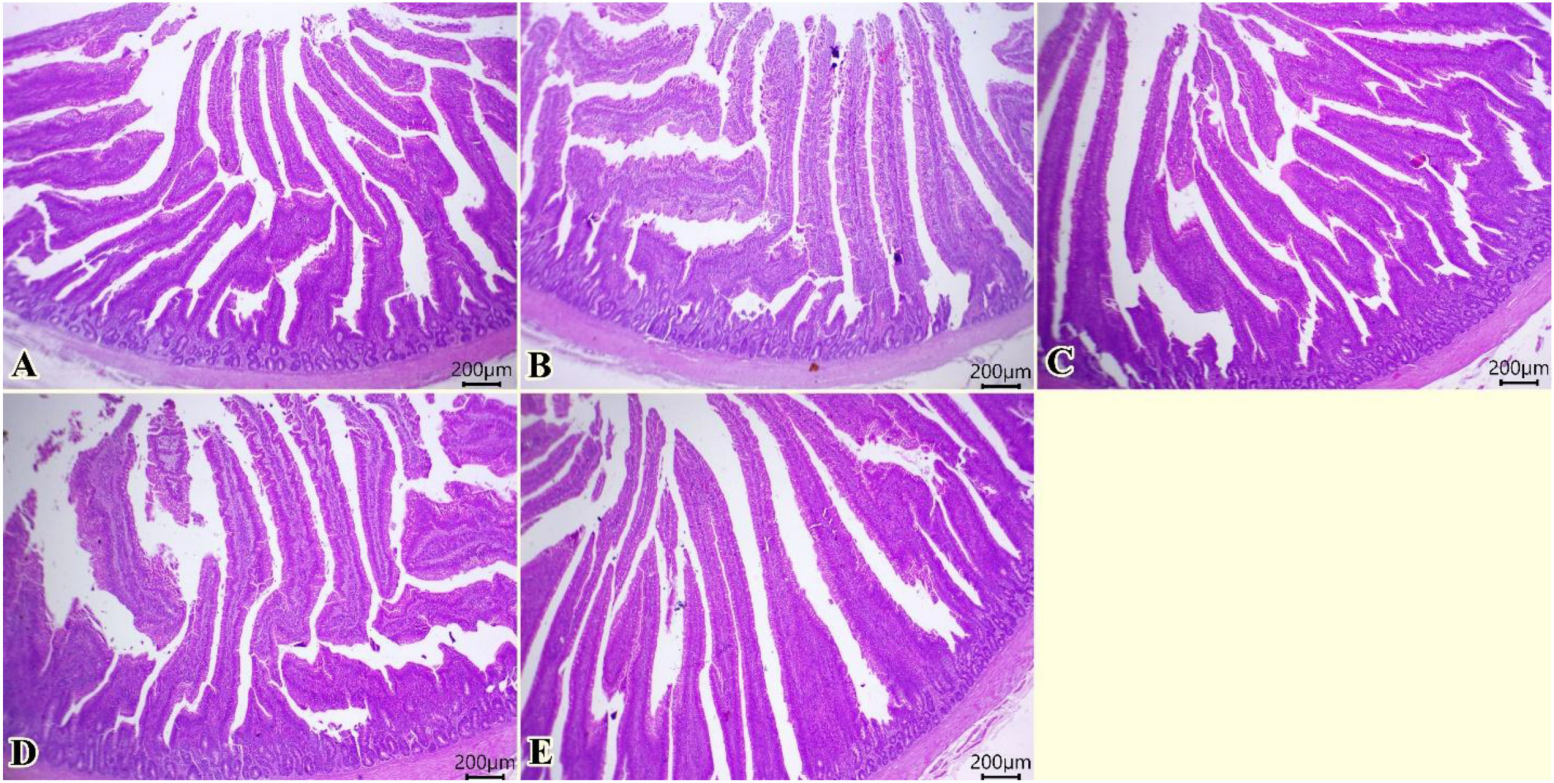
Photomicrograph of histopathological sections of the intestine **(A–E)** (scale bar 200 µm) stained with hematoxylin and eosin showing normal architectures of columnar enterocytes lining the mucosal villi, submucosal layer, and muscular layer with enhanced morphological structures of intestinal layers primarily villi. Panels **(A–E)** correspond to 0, 75, 150, 225, and 300 mg/kg of thyme oil, respectively.

Normal architectures of columnar enterocytes lining the mucosal villi, submucosal layer, and muscular layer were seen in all examined groups (**Figure 2**). Moreover, enhanced morphological structures of intestinal layers, primarily villi, were observed (0, 75, 150, 225, 300 of TO mg/kg, respectively, in **Figures 2A–E**).

**Table 5** shows that dietary TO supplementation had a significant effect on VL and CD, with both parameters increasing progressively (*P* = 0.003 and *P* = 0.041, respectively) as the inclusion level rose from 150 to 300 mg TO/kg diet. Similarly, the ASA was significantly improved by TO supplementation, reaching its highest values (*P* = 0.043) at 225 and 300 mg/kg. In contrast, VW at the dose of 75 mg/kg of TO was not significantly influenced (*P* = 0.166) by dietary treatments (**Table 5**).

**Table 5:** Effect of dietary supplementation with varying concentrations of thyme oil on intestinal morphology of broiler chickens.

Parameters	Thyme oil level (mg/kg diet)					SEM	*P* value
	0	75	150	225	300		
Villus length (VL, µm)	1238 *d*	1373 *c*	1451 *bc*	1569 *a*	1652 *a*	44.37	0.003
Villus width (WV, µm)	186.0 *a*	196.3 *a*	201.0 *a*	206.3 *a*	207.0 *a*	6.211	0.166
Absorption surface area (ASA, mm^2^)	0.206 *d*	0.256 *c*	0.292 *b*	0.324 *ab*	0.342 *a*	0.017	0.043
Crypt depth (CD, µm)	176.7 *d*	184.7 *cd*	188.3 *c*	204.0 *b*	215.0 *a*	4.783	0.041
Villus length/crypt depth (VL/CD)	7.006 *c*	7.434 *a*	7.706 *a*	7.691 *a*	7.684 *a*	0.095	0.630

Values with the same letter within a row are not significantly (*P*>0.05) different according to Tukey’s test.

**Figure 3:**
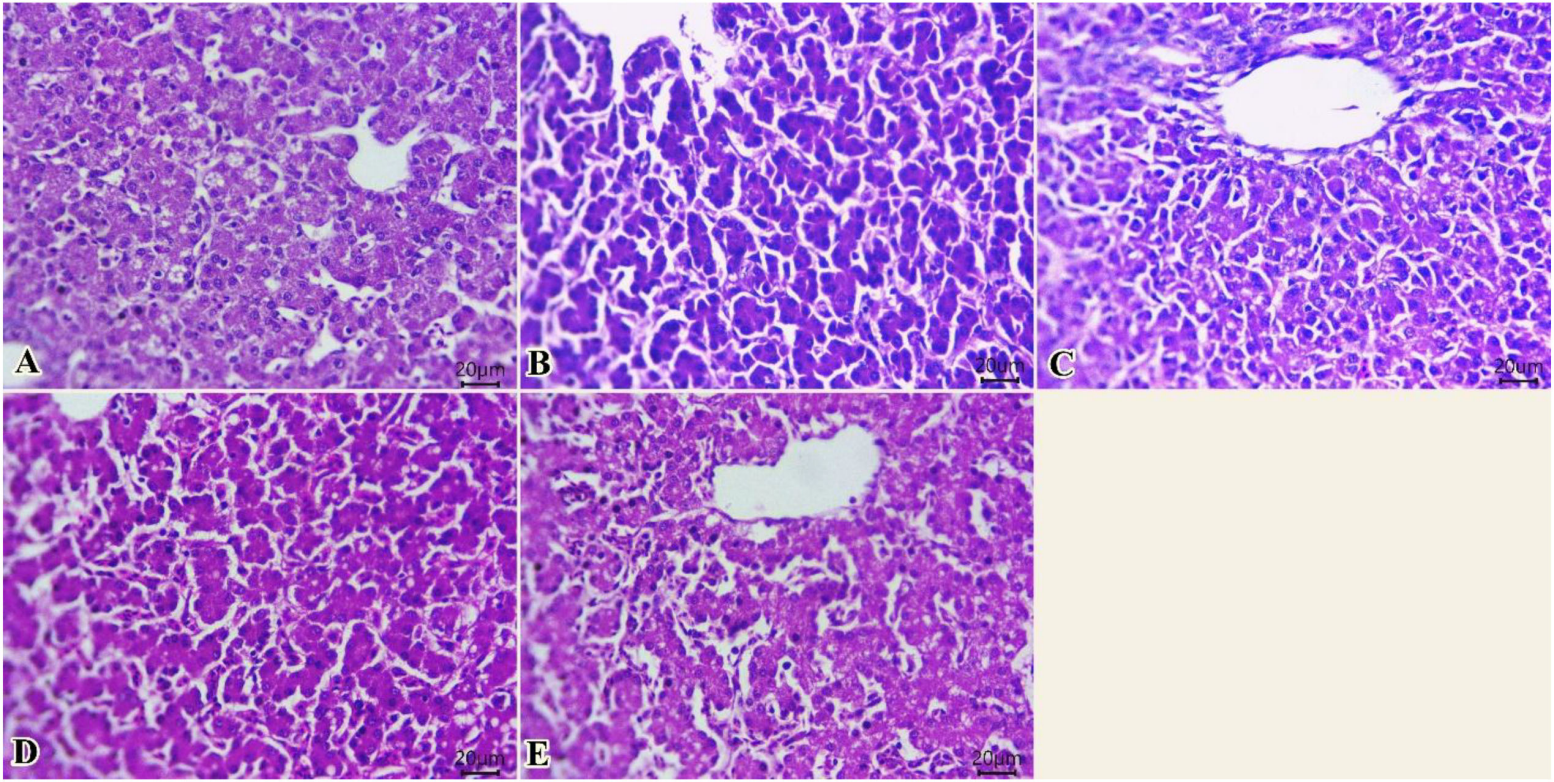
Photomicrograph of histopathological sections of the liver **(A–E)** (scale bar 20 µm) stained with hematoxylin and eosin showing normal histological structures of hepatic acini, Kupffer cells, sinusoids, and central veins with intracytoplasmic vacuolations with centrally located nuclei. Panels **(A–E)** correspond to 0, 75, 150, 225, and 300 mg/kg of thyme oil, respectively.

The liver of different groups (**Figures 3A–E**) showed normal histological structures of hepatic acini, Kupffer cells, sinusoids, and central veins. The hepatocytes were spherical cells with slightly acidophilic cytoplasm and centrally rounded vesicular nuclei. Intracytoplasmic vacuolations due to either glycogen or fat deposits, with centrally located nuclei, were seen in some examined sections (**Figure 3**).

**Figure 4:**
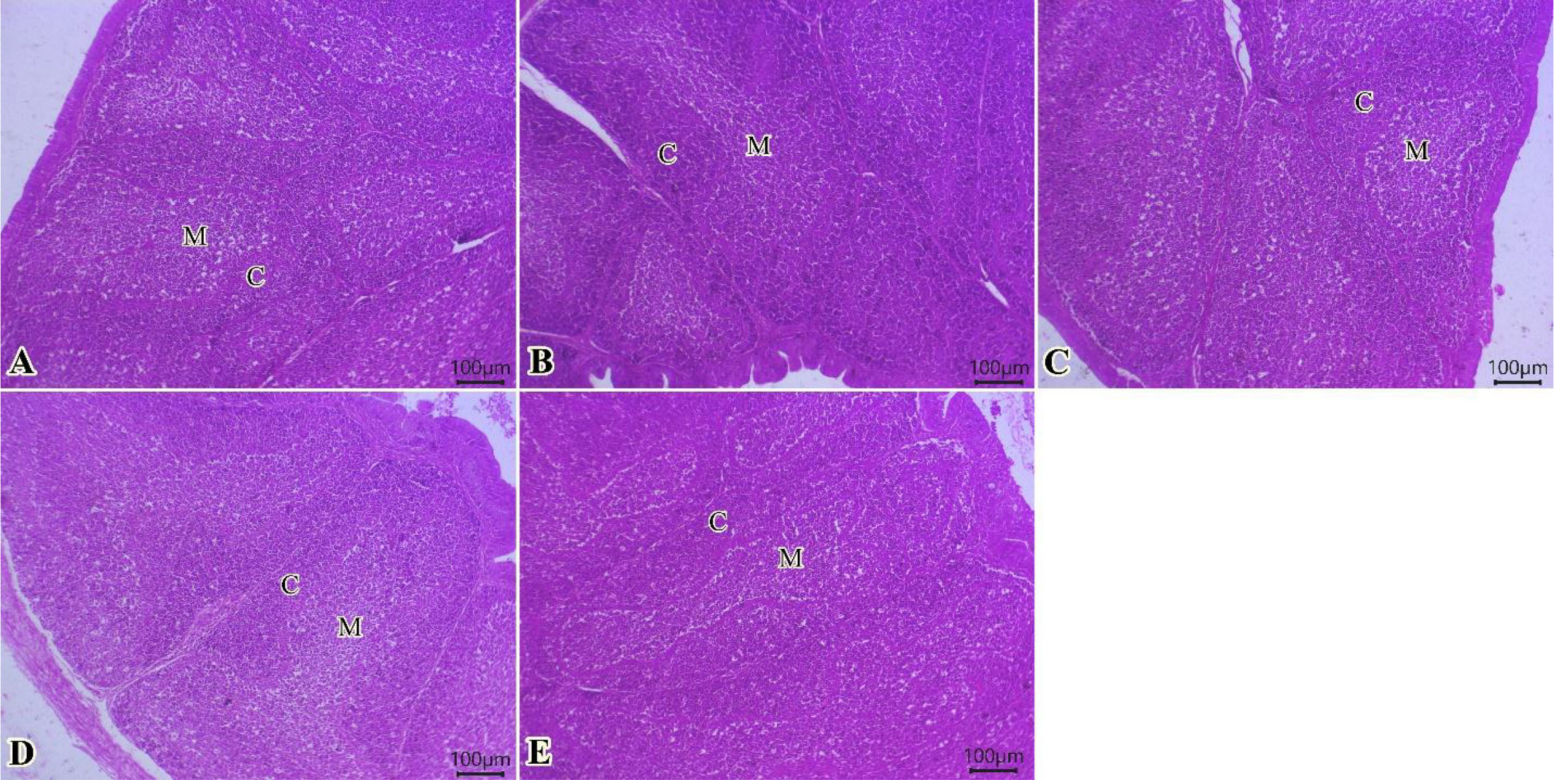
Photomicrograph of histopathological sections of the bursa **(A–E)** (scale bar 100 µm) stained with hematoxylin and eosin showing normal mucosal lining epithelium in addition to different sizes of cortical and medullary lymphoid follicles in all examined groups. Improvement in the activity of bursal lymphoid follicles, particularly in the medullary regions, was gradually observed. Panels **(A–E)** correspond to 0, 75, 150, 225, and 300 mg/kg of thyme oil, respectively. Cortical **(C)** and medullary (M) regions of bursal follicles.

The bursa of Fabricius (**Figures 4A–E**) showed normal mucosal lining epithelium in addition to different sizes of cortical and medullary lymphoid follicles in all examined groups. Improvements in the activity of bursal lymphoid follicles, particularly at medullary regions, were gradually seen (0, 75, 150, 225, and 300 of TO mg/kg) (**Figures 4A–E**).

**Figure 5:**
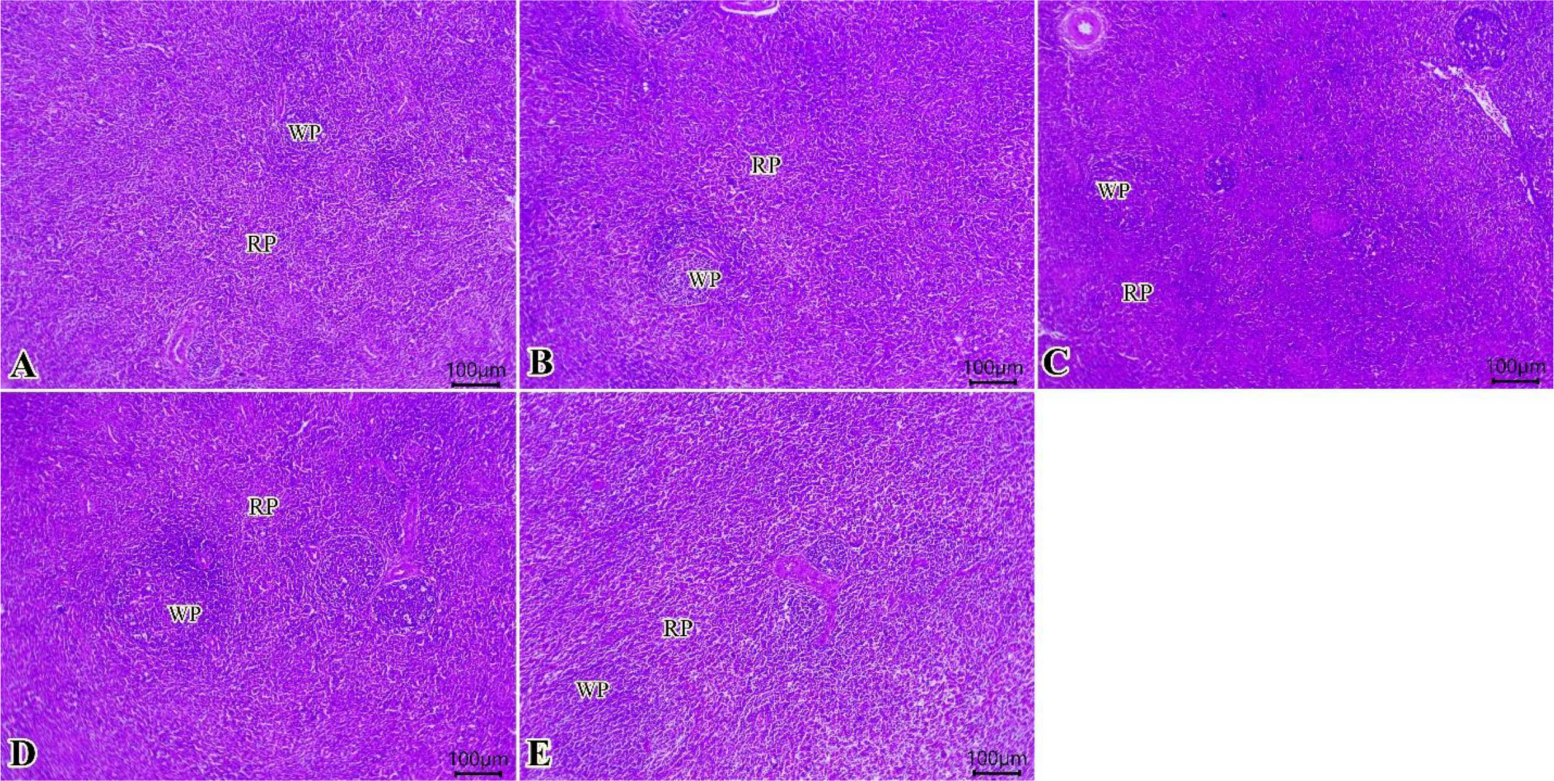
Photomicrograph of histopathological sections of the spleen **(A–E)** (scale bar 100 µm) stained with hematoxylin and eosin showing preserved histological architectures of white pulp and normal red pulp. A gradual increase in the contour of the white pulp lymphoid populations was observed. Panels **(A–E)** correspond to 0, 75, 150, 225, and 300 mg/kg of thyme oil, respectively. White pulp (WP) and red pulp (RP).

Sections from spleen from all examined groups (**Figures 5A–E**) showed preserved histological architectures of white pulp and normal red pulp that involve splenic sinusoids, a network of reticular fibers with lymphocytes, and macrophages. The increased contour of the lymphoid populations in the white pulp was gradually observed (0, 75, 150, 225, and 300 of TO mg/kg) (**Figures 5A–E**).

### Effect of dietary supplementation with TO on immune responses and hematological indices of broiler chickens

Among the tested immunoglobulins, IgA did not exhibit any statistically significant (*P =* 0.6998) changes due to the addition of 75 mg/kg of TO (**Table 6**). Significantly higher levels of IgM (*P* = 0.0066), IgG (*P* = 0.0323), and C3 (*P* = 0.0001) were induced by TO at 150, 225, and 300 mg/kg than control. TO at all doses significantly and dose-dependently increased lysozyme levels (*P* = 0.0043) compared with the control group (**Table 6**).

**Table 6:** Impact of dietary supplementation with varying concentrations of thyme oil on immunological parameters of broiler chickens.

Parameters	Thyme oil level (mg/kg diet)					SEM	*P* value
	0	75	150	225	300		
Spleen (%)	0.11 *c*	0.12 *c*	0.14 *b*	0.16 *a*	0.14 *b*	0.008	0.0155
Thymus (%)	0.46 *c*	0.52 *b*	0.58 *a*	0.51 *b*	0.53 *b*	0.017	0.0113
Bursa (%)	0.22 *c*	0.29 *b*	0.34 *a*	0.30 *b*	0.27 *b*	0.018	0.0098
IgM (mg/dl)	0.42 *c*	0.49 *bc*	0.65 *a*	0.59 *ab*	0.64 *a*	0.038	0.0066
IgA (mg/dl)	0.49 *a*	0.45 *a*	0.54 *a*	0.61 *a*	0.55 *a*	0.083	0.6998
IgG (mg/dl)	0.66 *c*	0.71 *bc*	0.90 *a*	0.95 *a*	0.91 *a*	0.063	0.0323
C3 (mg/dl)	42.25 *b*	48.31 *b*	85.23 *a*	77.93 *a*	80.33 *a*	4.435	0.0001
Lysozyme (mg/dl)	0.19 *d*	0.30 *c*	0.33 *bc*	0.37 *ab*	0.42 *a*	0.030	0.0043

Values with the same letter within a row are not significantly (*P*>0.05) different according to Tukey’s test. IgM, immunoglobulin M; IgA, immunoglobulin A; IgG, immunoglobulin G; C3, complement component 3.

Regarding the immune organ relative weights, the indices of spleen, thymus, and bursa were increased by all doses of TO inclusion (**Table 6**). Compared with the control birds, TO supplementation significantly enlarged the thymus index (*P =* 0.0113) at 75, 150, 225, and 300 mg/kg, the bursa index (*P =* 0.0098) at 75, 150, 225, and 300 mg/kg, and the spleen index (*P =* 0.0155) at 150, 225, and 300 mg/kg of TO (**Table 6**).

**Table 7** shows that the birds supplemented with TO at 150, 225, or 300 mg/kg diet presented significantly higher Hb (*P* = 0.0058), RBCs (*P* = 0.0070), and WBCs (*P* = 0.0279) than control birds. Hb, RBCs, and WBCs were most significantly (*P<0.05*) affected by 150, 225, and 300 mg/kg of TO in the feed (**Table 7**).

**Table 7:** Impact of dietary supplementation with varying concentrations of thyme oil on hematological parameters of broiler chickens.

Parameters	Thyme oil level (mg/kg diet)					SEM	*P* value
	0	75	150	225	300		
Hemoglobin (Hb) (g/dl)	7.52 *b*	7.56 *b*	7.92 *a*	8.10 *a*	8.09 *a*	0.102	0.0058
Red blood cells (RBCs) (10^6^/µL)	3.22 *c*	3.38 *c*	3.83 *ab*	4.08 *a*	3.72 *b*	0.129	0.0070
White blood cells (WBCs) (10^3^/µL)	20.42 *c*	22.28 *bc*	23.13 *ab*	23.32 *ab*	24.01 *a*	0.659	0.0279

Values with the same letter within a row are not significantly (*P*>0.05) different according to Tukey’s test.

### Effect of dietary supplementation with TO on liver and renal functions of broiler chickens

Among liver enzymes, AST was significantly decreased (*P* = 0.0002) by all TO doses, with a dose-dependent effect. The AST/ALT ratio was unaffected (*P =* 0.1768) by TO inclusion (**Table 8**). TO supplementation improved kidney function, as evidenced by reductions in creatinine and urea levels (*P* = 0.0154 and *P* = 0.0232, respectively) following TO administration.

**Table 8:** Effect of dietary supplementation with varying concentrations of thyme oil on liver and kidney functions of broiler chickens.

Parameters	Thyme oil level (mg/kg diet)					SEM	*P* value
	0	75	150	225	300		
(AST) (IU/L)	72.66 *a*	61.11 *b*	51.98 *c*	42.77 *d*	41.39 *d*	3.220	0.0002
(ALT) (IU/L)	15.85 *ab*	16.63 *a*	11.61 *c*	12.38 *c*	13.52 *bc*	1.210	0.0645
AST/ALT ratio	4.69 *a*	3.76 *a*	4.53 *a*	3.59 *a*	3.06 *a*	0.414	0.1768
Creatinine (mg/dl)	1.19 *a*	0.96 *b*	0.82 *bc*	0.74 *c*	0.81 *bc*	0.077	0.0154
Urea (mg/dl)	1.44 *a*	1.34 *a*	1.33 *a*	1.14 *b*	1.21 *b*	0.053	0.0232
Uric acid (mg/dl)	8.58 *a*	7.92 *a*	8.20 *a*	7.78 *a*	7.22 *a*	0.577	0.1523

Values with the same letter within a row are not significantly (*P*>0.05) different according to Tukey’s test. AST, aspartate aminotransferase; ALT, alanine aminotransferase.

A significant effect was observed at 75, 150, 225, and 300 mg/kg of TO for creatinine, and at 225 and 300 mg/kg for urea. In contrast, uric acid levels were not affected (*P* = 0.1523) by 75, 150, and 225 mg/kg of TO (**Table 8**).

### Effect of dietary supplementation with TO on the blood biochemical parameters of broiler chickens

Blood Mg, Ca, and Fe were increased (*P =* 0.0017, 0.0003, and 0.0033, respectively) by the dietary inclusion of TO, whereas P showed no statistical (*P =* 0.9313) difference (**Table 9**). Supplementation with TO at doses ≥150 mg/kg significantly increased Mg and Ca, and at 75 and 150 mg/kg, it significantly elevated Fe compared with the control (**Table 9**).

**Table 9:** Effect of dietary supplementation with varying concentrations of thyme oil on blood biochemical parameters of broiler chickens.

Parameters	Thyme oil level (mg/kg diet)					SEM	*P* value
	0	75	150	225	300		
Magnesium (Mg) (mg/dl)	5.13 *c*	5.41 *c*	5.82 *b*	6.08 *ab*	6.26 *a*	0.148	0.0017
Iron (Fe) (μmol/L)	18.69 *cd*	20.99 *b*	22.85 *a*	19.94 *bc*	18.52 *d*	0.612	0.0033
Calcium (Ca) (mg/dl)	8.10 *d*	8.69 *d*	10.18 *bc*	10.97 *a*	9.99 *c*	0.292	0.0003
Phosphorus (P) (mg/dl)	5.23 *a*	5.36 *a*	5.18 *a*	5.64 *a*	5.52 *a*	0.416	0.9313
Glucose (mg/dl)	368.35 *a*	352.34 *ab*	339.93 *b*	304.70 *c*	292.20 *c*	10.225	0.0018
Total protein (g/dl)	3.02 *b*	3.30 *b*	3.71 *a*	3.74 *a*	3.65 *a*	0.083	0.0007
Albumin (g/dl)	1.61 *a*	1.77 *a*	1.91 *a*	1.78 *a*	1.78 *a*	0.098	0.4288
Globulin (g/dl)	1.41*c*	1.53 *c*	1.80 *b*	1.96 *a*	1.88 *ab*	0.054	0.0001
Albumin/globulin (%)	1.15 *a*	1.16 *a*	1.07 *a*	0.91 *a*	0.95 *a*	0.067	0.1470

Values with the same letter within a row are not significantly (*P*>0.05) different according to Tukey’s test.

Blood mineral levels were most positively affected overall by a diet containing 150 mg/kg of TO. The inclusion of TO at ≥150 mg/kg increased total protein and globulin (*P =* 0.0007 and 0.0001, respectively), whereas albumin did not alter statistically (*P =* 0.4288).

The birds supplemented with TO at 75, 150, 225, or 300 mg/kg diet presented with lower glucose levels (*P =* 0.0018) than control birds. TO at 225 and 300 mg/kg diet significantly reduced glucose compared with 75 and 150 mg/kg of the diet (**Table 9**).

### Effect of dietary supplementation with TO on antioxidant parameters of broiler chickens

Dietary TO supplementation at 75, 150, 225, and 300 mg/kg significantly reduced oxidative stress, as indicated by decreased MDA levels (*P* = 0.0006) compared with the 0 mg/kg diets. Likewise, the levels of different antioxidants were comparable between birds that received TO at 0 and 75 mg/kg of the diet (**Table 10**).

**Table 10:** Impact of dietary supplementation with varying concentrations of thyme oil on antioxidant parameters of broiler chickens.

Parameters	Thyme oil level (mg/kg diet)					SEM	*P* value
	0	75	150	225	300		
SOD (U/mL)	0.25 *c*	0.29 *bc*	0.33 *ab*	0.37 *a*	0.36 *a*	0.026	0.0340
CAT (ng/mL)	0.24 *b*	0.31 *b*	0.41 *a*	0.38 *a*	0.40 *a*	0.022	0.0017
MDA (nmol/mL)	0.35 *a*	0.29 *b*	0.20 *c*	0.15 *c*	0.17 *c*	0.024	0.0006
TAC (ng/mL)	0.27 *d*	0.32 *cd*	0.36 *bc*	0.38 *ab*	0.41 *a*	0.022	0.0170
GSH (mg/dl)	0.25 *c*	0.33 *bc*	0.34 *b*	0.38 *ab*	0.41 *a*	0.029	0.0296
GST (mg/dl)	0.17 *a*	0.17 *a*	0.19 *a*	0.21 *a*	0.22 *a*	0.028	0.3173

Values with the same letter within a row are not significantly (*P*>0.05) different according to Tukey’s test. SOD, superoxide dismutase; CAT, catalase; MDA, malondialdehyde; TAC, total antioxidant capacity; GSH, reduced glutathione; GST, glutathione S-transferases.

The additions of 150–300 mg/kg of TO raised CAT (*P =* 0.0017) and TAC (*P* = 0.0170) levels, whereas dietary additions of 150, 225, and 300 mg/kg enhanced GSH (*P =* 0.0296) and SOD (*P =* 0.0340) levels compared with the 0 mg/kg diets (**Table 10**). Serum GST activity showed no statistical (*P* = 0.3173) differences across treatments (**Table 10**).

### Effect of dietary supplementation with TO on lipid profile of broiler chickens

**Table 11** shows that broiler chickens given dietary TO at all doses had significantly lower total cholesterol (*P* = 0.0015), triglycerides (*P* = 0.0008), LDL (*P* = 0.0001), VLDL (*P* = 0.0008), and LDL/HDL ratios (*P* = 0.0002), while having significantly higher HDL (*P* = 0.0134) only at 225 and 300 mg of TO/kg diet than non-supplemented birds (**Table 11**). Compared with the non-supplemented diet, the 300-mg/kg diet produced the highest HDL level, followed by the 225-mg/kg diet (**Table 11**).

**Table 11:** Effect of dietary supplementation with varying concentrations of thyme oil on lipid profile of broiler chickens.

Parameters	Thyme oil level (mg/kg diet)					SEM	*P* value
	0	75	150	225	300		
TC (mg/dl)	183.99 *a*	167.92 *b*	135.54 *c*	137.48 *c*	143.50 *c*	6.270	0.0015
TG (mg/dl)	87.98 *a*	72.32 *b*	53.06 *c*	57.21 *c*	51.02 *c*	4.451	0.0008
HDL (mg/dl)	41.76 *b*	45.83 *b*	47.97 *b*	56.03 *a*	59.48 *a*	3.065	0.0134
LDL (mg/dl)	124.64 *a*	107.62 *b*	76.96 *c*	70.01 *c*	73.82 *c*	5.596	0.0001
VLDL (mg/dl)	17.60 *a*	14.46 *b*	10.61 *cd*	11.44 *c*	10.20 *d*	0.891	0.0008
LDL/HDL ratio	3.02 *a*	2.39 *b*	1.60 *c*	1.25 *d*	1.24 *d*	0.141	0.0002

Values with the same letter within a row are not significantly (*P*>0.05) different according to Tukey’s test. TC, total cholesterol; TG, triglycerides; HDL, high-density lipoprotein; LDL, low-density lipoprotein; VLDL, very low-density lipoprotein.

### Effect of dietary supplementation with TO on carcass traits of broiler chickens

No significant changes were seen between birds receiving 75, 225, and 300 mg/kg of TO and those on non-supplemented diets regarding carcass percentage (*P* = 0.5762) (**Table 12**). Furthermore, no significant differences were observed between birds receiving 75 or 225 mg/kg of TO and those on non-supplemented diets for dressing (*P* = 0.4097), giblets (*P* = 0.1557), and heart (*P* = 0.3904) at 42 days of age. No significant differences were observed between birds receiving 75, 150, 225, and 300 mg/kg of TO and those on non-supplemented diets in liver percentage (*P* = 0.8092) at 42 days of age.

**Table 12:** Effect of dietary supplementation with varying concentrations of thyme oil on carcass traits and relative organs of broiler chickens.

Parameters (%)	Thyme oil level (mg/kg diet)					SEM	*P* value
	0	75	150	225	300		
Carcass	73.54 *a*	74.53 *a*	75.12 *a*	74.18 *a*	74.78 *a*	0.681	0.5762
Liver	2.32 *a*	2.23 *a*	2.37 *a*	2.37 *a*	2.33 *a*	0.086	0.8092
Gizzard	3.23 *c*	3.30 *bc*	3.67 *a*	3.50 *ab*	3.66 *a*	0.090	0.0252
Heart	0.54 *a*	0.59 *a*	0.65 *a*	0.59 *a*	0.60 *a*	0.035	0.3904
Giblets	6.09 *a*	6.12 *a*	6.69 *a*	6.46 *a*	6.58 *a*	0.171	0.1557
Dressing	79.62 *a*	80.65 *a*	81.81 *a*	80.63 *a*	81.63 *a*	0.752	0.4097

Values with the same letter within a row are not significantly (*P*>0.05) different according to Tukey’s test.

However, diets supplemented with 150 to 300 mg/kg of TO significantly increased gizzard percentage (*P* = 0.0252) compared with diets supplemented with 0 or 75 mg/kg of TO (**Table 12**).

### Effect of nutritional supplementation of TO on the economic assessment of broiler chickens

**Figure 6:**
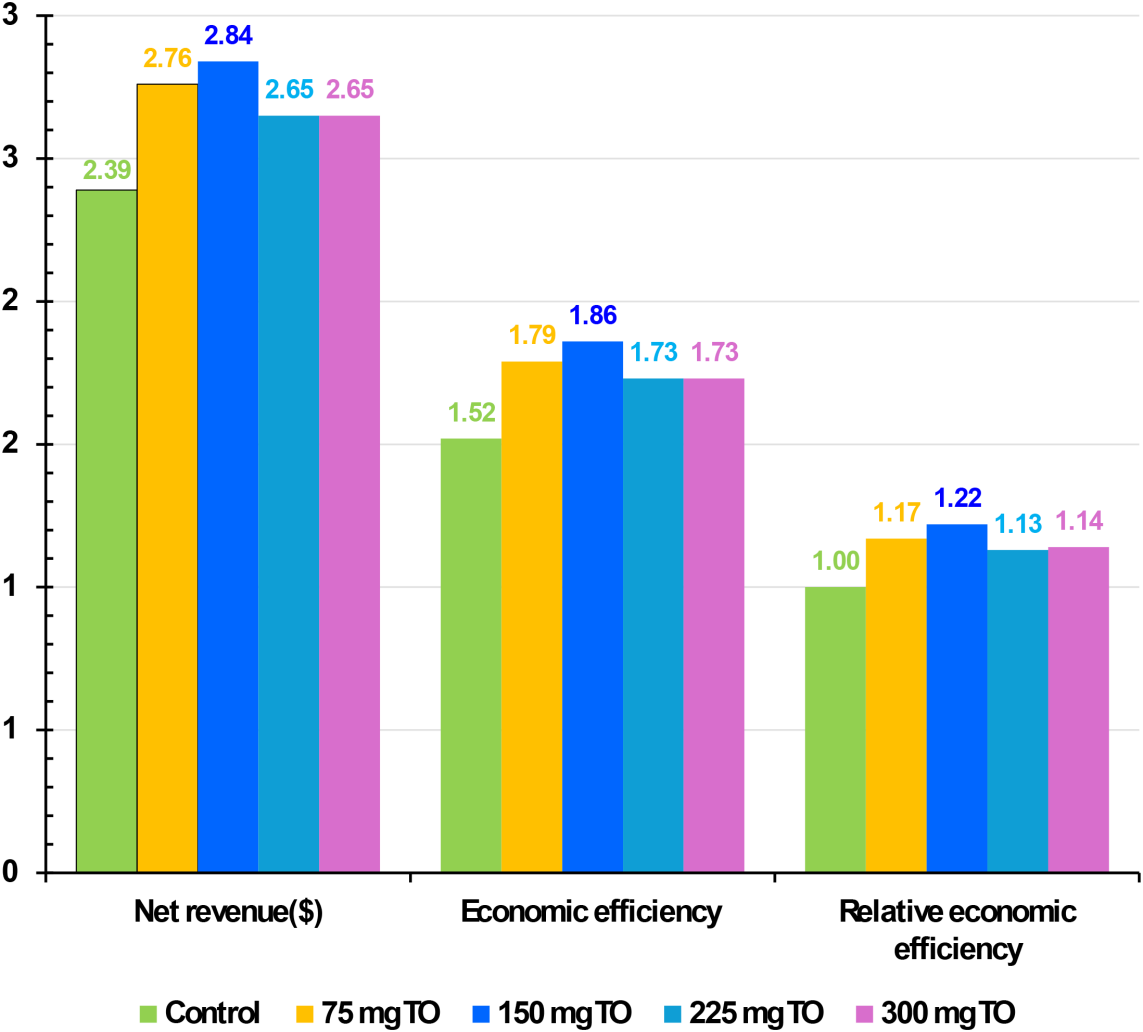
Effect of nutritional supplementation with different amounts of thyme oil (TO) on the economic assessment of broiler chickens.

**Figure 6** displays the impact of adding various quantities of TO to the diet of broiler chickens on NR, EE, and relative economic efficiency (REE) at 6 weeks of age. Higher economic indices (NR, EE, and REE) were achieved in broiler groups fed a diet containing 150 mg/kg (**Figure 6**).

This was followed by the broiler group receiving 75 mg/kg of TO, then both 225 and 300 mg/kg of TO (**Figure 6**). In contrast, the lowest values across all economic aspects were recorded in the control group (**Figure 6**).

## Discussion

The findings of several studies regarding the effect of TO supplementation on chicken growth performance were inconsistent. Several reports indicated no significant changes in BWG, FI, or FCR at 120 mg/kg (29), 0.05%–0.1% (30), or 1–2 g/kg, respectively (17). Similarly, diets containing 100–300 mg/kg TO did not alter BWG or FCR, although FI was enhanced at 100 and 200 mg/kg (18).

Shamma et al. (31) observed that 0.4 mL/kg TO reduced FI and improved FCR, despite no significant effect on BWG, whereas 300 mg/kg TO depressed both BWG and FI (32). The BWG and FCR results are consistent with those of Wade et al. (33) and Moustafa et al. (34), who reported that 100 mg/kg of TO significantly improved BWG and FCR.

In addition, the present study demonstrated that feeding broilers TO for 42 days significantly improved final BW, overall BWG, and FCR at all tested levels without affecting FI. These results agree with previous findings in broilers, quail, and ducks (35–38). The inconsistent results of the previous studies may be due to differences in the chemical composition of the oil, bird strains and other genetic backgrounds, housing and environmental conditions, management practices, or variations in experimental designs.

The animals’ metabolism, nutrition, and overall health were directly indicated using blood biochemical indices. This was demonstrated by the notable improvement in blood, protein, and mineral profiles. However, blood albumin levels were unaffected by TO supplementation at any dose (except 150 mg/kg), and only doses of 150, 225, and 300 mg/kg significantly reduced blood glucose (*P*<0.05). However, Noruzi et al. (39) had comparable results for albumin, showing that 250 mg/kg of thyme essential oil had no effect on blood albumin levels but did not affect blood glucose levels. In line with the present findings, adding dietary thyme from various forms—powder or oil—significantly improved WBCs, lymphocytes, and heterophils and raised hematological parameters (40). Consequently, in broiler chickens, thyme essential oils may improve protein metabolism and fortify the immune system (36, 41).

All TO levels improved liver function, with AST levels significantly reduced as the TO dose increased (*P*<0.05). Increased AST levels in animals and birds indicate degenerative liver disorders and alterations (42). A similar effect on the liver was observed by Moustafa et al. (34), where the serum activity of liver transaminases was significantly lower in birds fed TO at 100 mg/kg than in the controls. However, broilers fed TO at 100, 200, and 300 mg/kg showed insignificant variations in their blood activity of ALT and AST (18).

Furthermore, the current study showed that supplementing broiler chickens with TO for 42 days improved renal function by lowering creatinine, uric acid, and urea levels. These outcomes align with the findings of Gumus et al. (43), where the serum creatinine concentration of quails fed TO at 150, 300, and 450 mg/kg was decreased linearly in comparison with the non-supplemented group. Furthermore, feeding layers 6 and 9 g of thyme powder/kg diet significantly decreased the blood content of urea compared with the controls (44). Therefore, thyme essential oil can help broiler health, performance, and liver and kidney functions (45). However, in the present study, there was no significant effect on the percentage of carcass, liver, and heart due to TO feeding, which is consistent with that of Adam et al. (40).

Our findings showed that groups fed TO had significantly improved lipid profiles, consistent with those of Adam et al. (40). This effect could be related to the antioxidant capabilities of the thyme plant, as well as its capacity to inhibit the actions of specific lipases *in vivo*, regulate and control hormone levels, increase lipid catabolism, decrease fat deposition, and promote protein deposition in the body (41).

TO has antioxidant activity, in addition to immune-stimulating and antimicrobial properties, which help increase production efficiency. Oxidative stress is a significant factor affecting the general health and growth performance of chickens, and it is the imbalance between reactive oxygen species generation and antioxidant defense mechanisms (14). Therefore, oxidative stress was indicated by the elevated MDA and low levels of antioxidant activity (46).

The inclusion of TO in broiler chicken feed at a minimum dosage of 150 mg/kg in the current study improved some antioxidant parameters (SOD, CAT, TAC, and GSH) and significantly decreased lipid peroxidation (MDA). These results are consistent with those of Placha et al. (30) and AbdelGhaney et al. (47). The antioxidant activity correlates with the presence of phenolic compounds and the essential oil content (48).

The indices of the lymphoid organs (thymus, spleen, and bursa), the three main immunological organs in chickens, could be utilized to determine the immunological status (49). The bursa is more readily impacted by nutrition than the other lymphoid organs (50). The immune organ indices IgM, IgG, C3, and lysozyme were significantly increased by TO supplementation at 150, 225, and 300 mg/kg compared with the control. According to several studies, thyme extracts, oils, or powder strengthen the immune system by increasing the development of antibodies against avian viruses and the immunological response to viral vaccinations (1, 19, 41) and improving serum cytokine levels (INF-γ and IL-10) (47).

Generally, improvements in digestive system function, influenced by intestinal microbiota, histomorphology, and digestive enzymes, are linked to improved growth performance. The production of digestive enzymes (protease, amylase, trypsin, and lipase) was significantly increased on the 42^nd^ day with TO supplementation at 150, 225, and 300 mg/kg in the diet (**Table 3**). Birds supplemented with an equal mixture of thymol + carvacrol at levels 60, 100, and 200 mg/kg of feed, intestinal and pancreatic trypsin, lipase, and protease activities increased at 24 days old, but not at 42 days old (51). These elevated digestive enzyme levels in the current study may improve feed efficiency by improving the digestion and utilization of protein, fat, and carbohydrates.

Numerous studies have demonstrated how controlling gut bacteria can enhance growth performance (52, 53). In the present work, supplementing diets with TO at all tested levels (**Table 4**) significantly reduced pathogen counts, whereas lactic acid bacteria were increased. These findings align with those of Gheisar et al. (54), who reported that feeding broiler chickens a 0.075% essential oil mix (including TO) boosted the population of *Lactobacillus*. These effects are attributed to the phenolic components of TO, mainly thymol and carvacrol, which exert antibacterial action by disrupting bacterial membranes (55).

Zhu et al. (52) further reported that dietary plant extracts enriched with carvacrol and thymol improved cecal microbiota diversity and activated multiple metabolic pathways, including protein digestion, amino acid metabolism, and the citric acid cycle. Similarly, thymol supplementation altered microbial composition and metabolic profiles in piglets (56), leading to the production of short-chain fatty acids such as propionate and butyrate that enhance gut barrier function and confer anti-inflammatory benefits. It should be noted that the microbiological analyses in this study were limited to culture-based enumeration, which may not fully reflect the complexity of the cecal microbial community. Future studies could use 16S rRNA gene sequencing or metagenomic approaches to gain a deeper, more comprehensive understanding of gut microbiota dynamics.

The crypts and villi of the intestinal absorptive epithelium critically determine the intestinal capacity for nutrient absorption (57, 58). In the current study, significant increases in VL, VW, ASA, CD, and VL/CD were observed with 150, 225, and 300 mg/kg of TO (**Table 5**), indicating enhanced intestinal histomorphology.

Enhancing intestinal histomorphology, besides gut health, immune status, antioxidant capacity, liver and kidney functions, hematological parameters, digestive enzymes, and blood minerals through TO supplementation (150–300 mg/kg), improves nutrient utilization and growth performance, thereby reducing feed costs per unit of gain and increasing economic efficiency in broiler production. The third group (150 mg/kg diet) achieved the best values of NR and EE when compared with the other groups and the control. The TO can positively affect the economics of broiler chickens by improving BWG, FCR, and livability, thereby reducing the cost per kilogram of BWG. This is achieved through multiple mechanisms, including improving immune responses, stimulating intestinal enzymes, and possessing antioxidant properties. Furthermore, the specific economic benefits depend on the level, the presence of stressors like temperature, and general management practices.

## Conclusion

Providing broiler chickens with feed containing TO resulted in a growth-enhancing effect; however, FI remained essentially unchanged. Improvements in digestive enzyme activity, intestinal morphology, and modulation of cecal microbiota (reducing pathogenic bacteria whereas increasing lactic acid bacteria) may have contributed to enhanced growth performance. TO demonstrated antioxidant properties by reducing MDA levels and enhancing antioxidant enzyme activity, whereas immunological-stimulatory benefits were indicated by increased immune organ indices, immunoglobulin levels, Hb level, and the counts of RBC and WBC.

The lipid profile improved with increased HDL and decreased LDL, VLDL, total cholesterol, and triglycerides. Carcass characteristics, however, were not significantly affected. The net revenue and economic efficiency of treated broilers were improved with TO supplementation. While supplementation with 150 mg/kg of TO appeared to provide the most pronounced benefits under the present controlled experimental conditions, this recommendation should be considered cautiously, given limitations such as the study’s short duration and the absence of challenge trials. Further research is needed to confirm the optimal inclusion level under practical field conditions.

## Data Availability

The original contributions presented in the study are included in the article/supplementary material. Further inquiries can be directed to the corresponding author.
